# MicroRNA393 is involved in nitrogen-promoted rice tillering through regulation of auxin signal transduction in axillary buds

**DOI:** 10.1038/srep32158

**Published:** 2016-08-30

**Authors:** Xiang Li, Kuaifei Xia, Zhen Liang, Kunling Chen, Caixia Gao, Mingyong Zhang

**Affiliations:** 1 Key Laboratory of South China Agricultural Plant Molecular Analysis and Genetic Improvement & Guangdong Provincial Key Laboratory of Applied Botany, South China Botanical Garden, Chinese Academy of Sciences, Guangzhou 510650, China; 2 State Key Laboratory of Plant Cell and Chromosome Engineering, Institute of Genetics and Developmental Biology, Chinese Academy of Sciences, Beijing 100101, China

## Abstract

Rice tillering has an important influence on grain yield, and is promoted by nitrogen (N) fertilizer. Several genes controlling rice tillering, which are regulated by poor N supply, have been identified. However, the molecular mechanism associated with the regulation of tillering based on N supply is poorly understood. Here, we report that rice microRNA393 (OsmiR393) is involved in N-mediated tillering by decreasing auxin signal sensitivity in axillary buds. Expression analysis showed that N fertilizer causes up-regulation of OsmiR393, but down-regulation of two target genes (*OsAFB2* and *OsTB1*). *In situ* expression analysis showed that OsmiR393 is highly expressed in the lateral axillary meristem. OsmiR393 overexpression mimicked N-mediated tillering in wild type Zhonghua 11 (ZH11). Mutation of *OsMIR393* in ZH11 repressed N-promoted tillering, which simulated the effects of limited N, and this could not be restored by supplying N fertilizer. Western blot analysis showed that OsIAA6 was accumulated in both OsmiR393-overexpressing lines and N-treated wild type rice, but was reduced in the *OsMIR393* mutant. Therefore, we deduced that N-induced OsmiR393 accumulation reduces the expression of *OsTIR1* and *OsAFB2*, which alleviates sensitivity to auxin in the axillary buds and stabilizes OsIAA6, thereby promoting rice tillering.

Rice tillering (branching) is an important agronomic trait, as the number of tillers per plant determines the panicle number, which is a key factor of the rice grain yield^1^,^2^. The rice tiller represents a form of shoot branching that only exists in monocotyledonous plants at the jointing stage. Unlike in *Arabidopsis*, the dominance of the apical meristem in rice is weak, so tillering occurs during the vegetative stage upon inhibition by the apical meristem or in previously activated buds, which is not sufficiently strong. However, when the panicles of the main stems begin to differentiate, the buds formed at the elongated upper internodes become dormant^3^. The dormant bud is activated and later transformed into an activated, growing tiller. This process is complicated and intricate. Rice tillering is mediated by the interplay of the environment and endogenous signals such as phytohormones. It is already known that auxin, cytokinin (CK), and strigolactones (SLs) are specific regulators involved in bud outgrowth regulation^4^. Auxin and SLs inhibit bud outgrowth, whereas CK activates and promotes this process^5^,^6^. Nitrogen (N) can considerably increase CK levels in rice tiller buds and nodes, and can augment IAAs (early auxin responsive genes) in rice nodes^7^. The tiller number per plant is strongly affected by N fertilizer availability. High N fertilizer allows rice to produce more tillers^7^, and the *Arabidopsis* root system architecture is mediated by N availability^8^.

More is known of the mechanism by which limited N reduces branching than how abundant N promotes branching. In *Arabidopsis*, nitrate limitation reduces shoot branching by both inhibiting bud initiation and weakening the basipetal sequence of bud initiation that is caused by flowering^9^. Auxin was the first phytohormone to be identified as an important regulator of plant branching. Bud outgrowth is strongly inhibited by the apex of a whole plant. If the apex is removed, previously inactivated axillary buds become active and plant branching is initiated^10^. However, auxin cannot enter axillary meristem cells and therefore, its inhibitory functions are deemed to be indirect^11^. Auxin suppresses bud development through at least two different processes. First, auxin is synthesized in the plant apex and is transported basipetally towards the root, which is known as polar auxin transport (PAT), and this inhibits bud outgrowth^12^. Because auxin cannot enter the axillary meristem cells, the second mechanism is regarded as the regulation of other signalling molecules (CK and SLs) by auxin; these proteins can enter axillary meristem cells and regulate axillary bud initiation and outgrowth^13^,^14^. The PAT model suggests that polar auxin transport and high auxin concentrations inhibit axillary bud development^14^,^15^. The second messenger model suggests that auxin regulates the distribution and activity of CKs and SLs in the axillary meristem to control plant branching. Both of these models are supported by recent studies^16^.

Several genes have been identified as regulators that control rice tillering and branching. *MOC1*^17^ controls initiation and outgrowth of axillary meristems at both the vegetative and reproductive stages. *LAX1* is a regulator that controls axillary meristem initiation and/or maintenance during rice reproductive development^18^. *OsMADS57* was reported to interact with *OsTB1*, and targets *D14* (Dwarf 14) to control the outgrowth of axillary buds^19^. OsmiR156 targets *OsSPL14* and mediates its degradation to control rice tillering^20^. In *Arabidopsis*, auxin and SL signalling are required to coordinate shoot branching with respect to N supply^9^.

MicroRNAs (miRNAs) are a class of oligonucleotides, 20 to 24 nucleotides long, and are endogenous small RNAs that are involved in post–transcriptional gene regulation in multicellular organisms, through influencing both the stability and translation of mRNAs^21^. miRNAs have important regulatory roles in the uptake, assimilation, and translocation of nutrients in plants^22^. miR395 and miR399 can regulate the distribution and homeostasis of sulphur and phosphate, respectively, in *Arabidopsis* and rice^23^,^24^. miR169 has specific roles in the plant’s response to N deficiency^25^. miR167 and miR393 were reported to regulate NO_3_^−^ signalling during lateral root development^8^,^26^. *MIR393* is conserved among different plant species^27^, and recent studies revealed that it has multiple functions in plant growth and development, such as controlling root architecture^8^, regulation of leaf development^28^, antibacterial resistance to pathogen attack^27^, tolerance to stress^29^, and maintenance of normal plant growth^30^. In *Arabidopsis*, miR393 is encoded by two distinct loci: *MIR393a* and *MIR393b*. In aerial tissues such as leaves, miR393 is mainly transcribed from *MIR393b*, suggesting distinct roles for *MIR393a* and *MIR393b*^28^. Moreover, miR393-guided target cleavage processes generate secondary small interfering RNAs (siRNAs) from miR393 target transcripts. Feedback regulates the targeting of neo-generated siRNA, guiding the cleavage of miR393 targets^28^. Similar to that in *Arabidopsis*, the rice OsmiR393 family is encoded by two loci, *OsMIR393a* and *OsMIR393b*^31^,^32^. Rice *OsMIR393a* and *OsMIR393b* also showed different expression patterns, which suggests that conserved mechanisms were adopted in monocots and eudicots during plant development^32^. To date, the exact mechanism of rice tillering regulation by OsmiR393 has not been reported.

miR393 was shown to target auxin (IAA) receptor genes *TIR1* and *AFB* in different plants, including *Arabidopsis* and rice^31^,^33^,^34^. *TIR1* and *AFB*s encode F-box proteins, which combine with three other proteins, ASK1, CUL1, and RBX, for assembly into the ubiquitin degradative complex (SCF^TIR1^) to degrade specific substrates during auxin signaling^35^,^36^,^37^. AUX/IAAs represent a class of proteins that represses auxin signalling. AUX/IAA proteins can bind to and repress ARFs (auxin response factor) to activate downstream auxin-responsive genes. TIR1 and AFBs recognize and bind IAA and degrade AUX/IAA proteins via the SCF^TIR1^ complex^37^ to ensure correct auxin signalling.

Here, we present findings showing that N-induced rice tillering is caused by attenuating the sensitivity of tiller buds to auxin through microRNA OsmiR393-mediated auxin signal transduction.

## Results

### Nitrogen supply promotes OsmiR393 accumulation in rice

**Figure 1:**
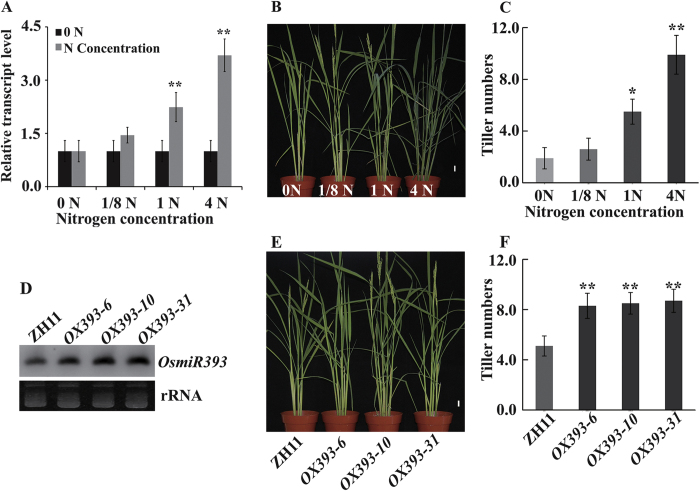
Nitrogen fertilizer triggers OsmiR393 accumulation and overexpression resulting in an increased number of tillers. (**A** and **D)**, qRT-PCR (**A**) and small RNA gel blot (**D**) analysis of OsmiR393 expression level in ZH11 with elevating NH_4_NO_3_ levels (**A**) and in OsmiR393-overexpressing rice lines (*OX393*) under normal nitrogen (N) levels (1 N) (**D**). The level of NH_4_NO_3_ (1.43 mM) in the IRRI nutrient solution^38^ was set as the normal (1 N) level of N fertilizer; other N levels represent multiples of the 1 N level. Vertical bars in (**A**) indicate standard error from three individual repeats. Rice *U6* was used as an internal control. The asterisk in (**A**) indicates a mean fold change of greater than 2 *vs* the 0 N condition. (**B**,**C)** and **(E**,**F)**, Tiller numbers of ZH11 with elevating NH_4_NO_3_ levels (**B,C**) and in OsmiR393-overexpressing rice plants with a normal N concentration (1 N) (**E**,**F**) respectively, when plants were grown in a pot. Scale bar = 2 cm in (**B**) and (**E**). Tiller numbers in (**C**) and (**F**) were counted and analysed in excel. Vertical bars in (**C**) and (**F**) indicate standard error (n ≥ 15 plants for every sample and each experiment was repeated three times). The asterisk indicates significant differences (*P ≤ 0.05 and **P ≤ 0.01) compared to ZH11 at 0 N (**C**), and at 1 N (**F**) as determined by a *t*-test.

In *Arabidopsis*, miR393, which targets *AFB3*, is a unique nitrogen (N) responsive module that mediates root system architecture in response to external and internal N availability^8^. To investigate whether OsmiR393 expression also responds to exogenous N fertilizer in rice, we analysed the accumulation of OsmiR393 with various levels of NH_4_NO_3_ fertilizer as the N source. The level of NH_4_NO_3_ (1.43 mM) in the IRRI nutrient solution^38^ was set to the normal level (1 N) of N fertilizer. OsmiR393 accumulation increased following exposure to elevating levels of N fertilizer, from 0 mM (0 N) to 5.72 mM (4 N) NH_4_NO_3_, as assessed by a quantitative reverse transcription polymerase chain reaction (qRT–PCR) (Fig. 1A) and by a small RNA gel blot (Supplemental Fig. S1). Under conditions of 1 N and 4 N NH_4_NO_3_, OsmiR393 expression increased by 1.4- and 3.5-fold, respectively, compared to that of the 0 N condition (Fig. 1A). These results showed that OsmiR393 accumulation in rice is induced by high N fertilizer levels, similar to that observed in *Arabidopsis*^8^.

### Overexpression of OsmiR393 mimics N-promoted rice tillering

High N fertilizer can promote tillering in rice, and we found that OsmiR393-overexpressing rice produced more tillers than wild type rice with normal levels of N fertilizer^39^. Given that high N fertilizer promotes OsmiR393 accumulation (Fig. 1A, Supplemental Fig. S1), we hypothesized that OsmiR393 is involved in mediating N-mediated rice tillering. To test this hypothesis, we first measured the tiller number in wild type *japonica* rice cultivar Zhonghua 11 (ZH11) with different levels of N fertilizer (Fig. 1B,C). Tiller numbers in ZH11 were increased with elevating levels of NH_4_NO_3_. When grown under low N conditions, specifically, 0 and 0.18 mM NH_4_NO_3_ (0 N and 1/8 N, respectively), ZH11 produced 2.6 tillers on average. At normal N levels (1.43 mM NH_4_NO_3_; 1 N), it produced 5.5 tillers, which is 2-fold greater than that of the 0 N condition. The tiller number reached approximately 10 when plants were cultivated with high N (5.72 mM NH_4_NO_3_; 4 N), which is 4-fold greater than that of the 0 N condition. Furthermore, tillering was analysed in three OsmiR393-overexpressing rice lines^39^, relative to N content. A small RNA gel blot assay confirmed that OsmiR393 accumulated in the three lines (*OX393-6*, *OX393-10*, and *OX393-31*) compared to expression in wild type ZH11, under normal N conditions (1 N) (Fig. 1D). When grown under normal N (1 N), all three OsmiR393-overexpressing lines produced more tillers than ZH11 (Fig. 1E,F). At 1 N, tiller numbers in the three OsmiR393-overexpressing lines reached approximately 8.3, which was about 1.5-fold higher than that observed in ZH11. These results demonstrate that tillering with OsmiR393 overexpression at normal N levels mimics that of ZH11 at higher N levels. Third, to investigate whether OsmiR393 overexpression at different N levels increases tillers as in ZH11, we treated *OX393-6* with a gradient of N levels (Supplemental Fig. S2A,B). Although the tiller number in *OX393-6* increased with elevating N levels, it did not reach the maximum observed with ZH11 (Fig. 1B,C). With 4 N conditions, the tiller number reached 7.4 in ZH11, but averaged only 5 in *OX393-6*. Moreover, at each N level (except 4 N), *OX393-6* produced more tillers than ZH11. However, *OX393-6* and ZH11 reached approximately the same tiller numbers when they were grown at 4 N. At the 1/8 N level, *OX393-6* produced on average 5.2 tillers, whereas ZH11 produced 3.8 tillers. At the 1 N level, *OX393-6* produced on an average 6.8 tillers and ZH11 produced 4.4 tillers. At the 4 N level, the tiller number for both *OX393-6* and ZH11 reached approximately 9.5. In summary, our data suggests that OsmiR393 overexpression is sufficient to increase tillering at almost all N concentrations, indicating that it is involved in N-promoted tillering. However, OsmiR393 overexpression cannot fully mimic N-induced tillering at 4 N, suggesting that other factors/signalling pathways are involved in this process.

### N fertilizer cannot restore the reduced tiller phenotype of an *OsMIR393* mutant

**Figure 2:**
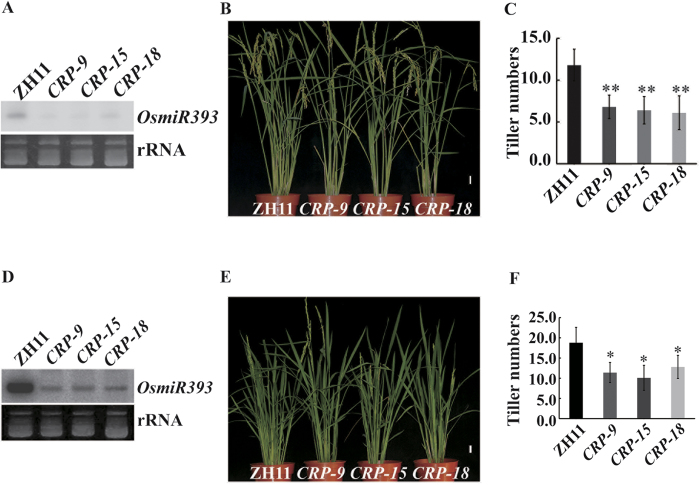
*OsMIR393* mutants develop fewer tillers (**A–C**) under 1 N condition and external application (4N) of NH_4_NO_3_ does not restore tillers in *OsMIR393* mutants (**D–F**). (**A** and **D)**, Small RNA gel blot analysis of OsmiR393 expression in *OsMIR393* mutants under normal nitrogen (N) levels (1 N) (**A**) and with 4 N levels of N fertilizer (**D**). (**B** and **C)** Tiller numbers for the *OsMIR393* mutant grown in a controlled field and supplied with 1 N fertilizer (NH_4_NO_3_). (**E** and **F)** Tiller numbers of *OsMIR393* mutants grown in a controlled field and supplied with 4 N fertilizer (NH_4_NO_3_). Vertical bars in (**C**) and (**F**) indicate standard error (n ≥ 15 plants for every sample and each experiment was repeated three times). The asterisk indicates significant differences (*P ≤ 0.05 and **P ≤ 0.01) compared to the ZH11 control, as assessed by a *t*-test. Scale bar = 3 cm in (**B**) and (**E**).

To confirm that OsmiR393 is involved in N-mediated rice tillering, *OsMIR393* mutants were generated using a clustered regularly interspaced short palindromic repeats (CRISPR) approach^40^,^41^. An sgRNA sequence was designed to target the mature *OsmiR393* sequence and ultimately disrupt this gene. Next, the sgRNA was linked to the sgRNA-Cas9 vector^42^ and transformed into ZH11. Three knock-out *OsMIR393* mutant lines were selected and designated as *CRP-9*, *CRP-15*, and *CRP-18*. A small RNA gel blot assay showed that OsmiR393 accumulation was significantly decreased (Fig. 2A). To inspect the tillering phenotype in these *OsMIR393* mutants, we grew them in a controlled paddy that was managed conventionally. Contrary to OsmiR393 overexpressing lines, which produced more tillers (Fig. 1E,F), *OsMIR393* mutants produced fewer tillers than ZH11 (Fig. 2B,C). ZH11 produced on average 12.2 tillers, whereas all *OsMIR393* mutants (*CRP-9*, *CRP-15*, and *CRP-18*) produced approximately 6–7 tillers, approximately half that of ZH11. Combining these results, we concluded that OsmiR393 regulates rice tillering. To investigate whether OsmiR393 is associated with N fertilizer in rice tillering, and if tillering could be restored in *OsMIR393* mutants by N fertilizer, we grew *OsMIR393* mutants at a 4 N fertilizer level (Fig. 2E,F). *OsMIR393* mutants were planted in a controlled paddy with the aforementioned fertilizer level. A high N level (4 N) did not restore OsmiR393 accumulation in *OsMIR393* mutants (Fig. 2D), and the tiller number in ZH11 was still much higher than that in *OsMIR393* mutants (Fig. 2E,F). This indicated that N fertilizer and OsmiR393 have overlapping functions in rice tillering. However, tiller numbers in *OsMIR393* mutants grown at 4 N were still much higher than in those grown at 1 N. *OsMIR393* mutants produced on average 11 tillers at the 4 N level (Fig. 2F), and produced approximately 6 tillers at the 1 N fertilizer level (Fig. 2C). This implies that OsmiR393 is required, but not sufficient, for N-mediated rice tillering. Thus, there could be some other mechanisms, distinct from OsmiR393, involved in rice tillering in the presence of N.

### Auxin signal transduction, mediated by OsmiR393, is involved in N-mediated rice tillering

**Figure 3:**
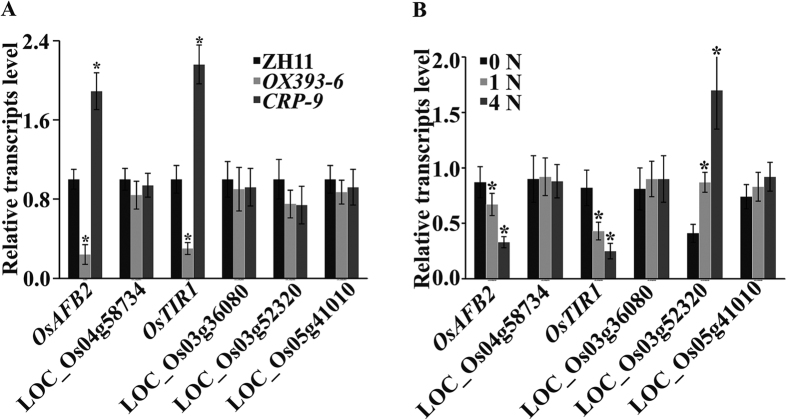
Inverse expression of OsmiR393 and its target genes (*OsAFB2* and *OsTIR1*) with elevating nitrogen (N) levels. (**A**) qRT-PCR verification of OsmiR393 targets in OsmiR393 overexpressing line (*OX393-6*), and *OsMIR393* mutant (*CRP-9*) with 1 N fertilizer. (**B**) qRT*–*PCR expression analysis of putative OsmiR393 target genes in wild type rice ZH11 with elevating N levels. Results are presented as the means of three experiments. Vertical bars indicate standard error. The asterisk mark indicates a mean fold change greater than 2 or less than 0.5 between ZH11 and the mutants (**A**), or the elevating N treated ZH11 (**B**).

Two auxin receptors *OsAFB2* and *OsTIR1* have been verified as target genes of OsmiR393, and seven genes were predicted to be candidate OsmiR393 targets in rice^32^,^39^. To better understand the actual targets of N fertilizer involved in rice tillering, plant materials were collected comprising regions between the shoot and root containing the SAM (shoot apical meristem), an area of the plant where tillers are formed, for RNA extraction and qRT–PCR detection. qRT-PCR results showed that *OsAFB2* and *OsTIR1* were down-regulated in the tillering region of *OX393-6*, and dramatically up-regulated in *CRP-9*, when compared to levels in ZH11, at N levels of 1 N (Fig. 3A).

To determine whether *OsAFB2* and *OsTIR1* also decreased with elevating N levels, qRT-PCR assays were conducted in wild type ZH11 (Fig. 3B). In contrast to over-expression of OsmiR393 with elevating N levels (Fig. 1A), *OsAFB2* and *OsTIR1* transcripts decreased (Fig. 3B). This demonstrated that the two target genes (*OsAFB2* and *OsTIR1*) and OsmiR393 are reciprocally expressed with elevating N levels, indicating that *OsAFB2* and *OsTIR1* are involved in the response to N supply in rice. However, the expression of another putative target gene (LOC_Os03g52320) was up-regulated in response to high N fertilizer levels in ZH11, suggesting that it was not a target of OsmiR393. LOC_Os03g52320 was predicted to encode a GRF1-interacting factor involved in various aspects of tissue differentiation and organ development^43^,^44^. We speculated that its expression was enhanced, either directly or indirectly, by N fertilizer to control rice seedling growth. One putative target, LOC_Os10g39790 was not amplified by PCR, indicating that it was not expressed in the region that we collected.

During auxin signal transduction, TIR1 and AFB2 perceive the auxin signal and degrade repressors of auxin signalling via the SCF (SKP1-CULLIN/cdc53-F-box) complex mediated by the 26S proteasome^45^. OsmiR393 overexpression and high levels of N, with OsmiR393 accumulation, might desensitize rice plants to exogenous auxin. In contrast, *OsMIR393* mutants should become hypersensitive under these conditions. To test this hypothesis, we treated ZH11, *OX393-6*, and *CRP-9* with NAA and observed their responses. The application of 5 mg L^–1^ NAA typically inhibited the outgrowth of rice tiller buds (Supplemental Fig. S3A, ZH11, and ZH11 mock). However, *OX393-6* and 4 N-treated ZH11 plants showed obvious resistance. Tiller buds in *CRP-9* were dramatically inhibited, exhibiting hypersensitivity (Supplemental Fig. S3A). This result further confirmed N-mediated rice tillering resulting from attenuated auxin signalling through OsmiR393-mediated cleavage of *OsTIR1*/*OsAFB2*. The callus induction rate is an important index reflecting auxin/cytokinin cross-interactions^46^. To further substantiate our hypothesis, ZH11, *OX393-6*, and *CRP-9* plants were induced to form calluses, and their callus induction rates were compared (Supplemental Fig. S3B). Calluses were induced in ZH11 at a faster rate than in OsmiR393-overexpressing lines, but at a slower rate than in the *OsMIR393* mutants. Moreover, adventitious root development is a typical process involved in auxin signaling^32^,^47^. The OsmiR393-overexpressing line, *OX393-6*, showed a significant reduction in adventitious roots, whereas that in *CRP-9* increased (Supplemental Fig. S3C,D). The above results implied that auxin signalling interferes with adventitious root formation in OsmiR393-overexpressing lines, but is attenuated in *OsMIR393* mutants.

### N fertilizer supply and OsmiR393 overexpression promote axillary bud outgrowth

**Figure 4:**
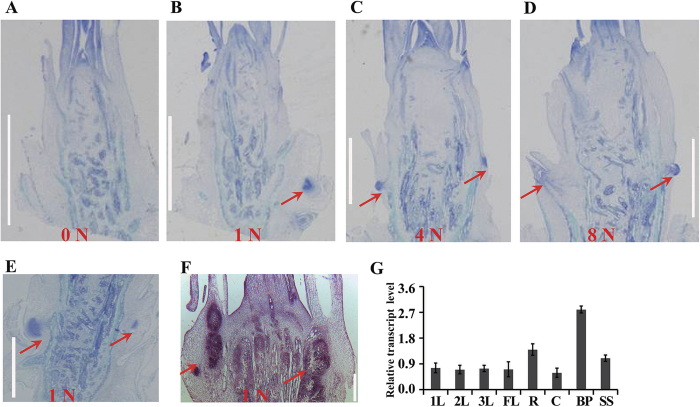
OsmiR393 affects axillary bud outgrowth and is expressed at a high level in the lateral axillary meristem. (**A**–**D**) Tissue section of wild type ZH11 rice grown with different nitrogen (N) levels using an NH_4_NO_3_ fertilizer. (**E**) Tissue section of OsmiR393-overexpressing line (*OX393-6*) under normal N levels (1 N) using NH_4_NO_3_ fertilizer. Red arrows in (**A–E**) indicate the formed axillary meristem. Pictures are representative of sections from 20 plants. (**F**) *In situ* hybridization of OsmiR393 in the ZH11 stem base with 1 N NH_4_NO_3_ fertilizer. The red arrow in (**F**) indicates intense OsmiR393 expression. Bar = 100 μm in (**A**–**F**). (**G**) qRT–PCR detection of OsmiR393 levels in different organs of ZH11 under normal N levels (1 N) using NH_4_NO_3_ fertilizer. Vertical bars indicate standard error from three repeats. 1L, the first leaf; 2L, the second leaf; 3L, the third leaf; FL, flag leaf; R, root; C, culm; BP, booting panicle; SS, shoot sheath.

**Figure 5:**
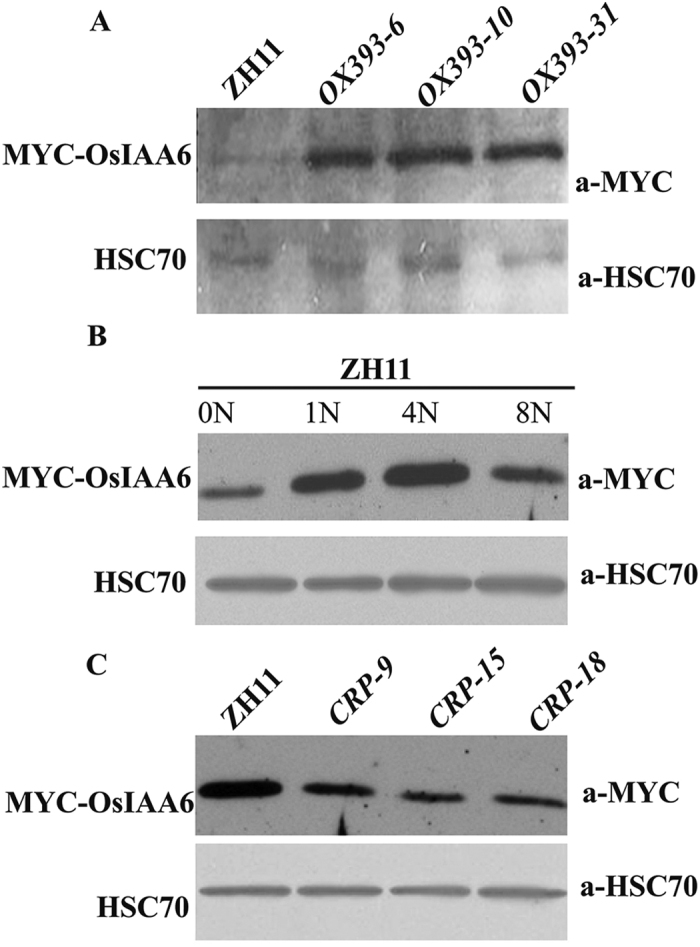
OsIAA6 protein level in ZH11 plants with different nitrogen (N) levels and in different transgenic rice with 1 N levels. (**A***–***C)** Western blot analysis of OsIAA6 in the protoplast of OsmiR393-overexpressing lines (*OX393*) (**A**) N-treated ZH11 (**B**) and *OsMIR393* mutants (*CRP*) (**C**). Protoplasts were prepared from 2-week old seedlings. HSC70 was used as a loading control.

To further investigate the relationship between OsmiR393 overexpression and N-mediated rice tillering, we explored the axillary bud formation process in ZH11 in response to different N levels and in the OsmiR393-overexpressing line, *OX393-6*, by tissue section (Fig. 4A–E; Supplemental Table S2). After 12 d of cultivation with different levels of N fertilizer, the axillary meristem numbers in ZH11 were inspected. No axillary meristems were observed when ZH11 was grown without N fertilizer (0 N, Fig. 4A), one was observed when ZH11 was grown at 1 N (Fig. 5B), and two were found with 4 N fertilizer (Fig. 4C). With an excessive N level (8 N), only one axillary meristem was observed, while the other had already formed an axillary bud (Fig. 4D). In contrast, the axillary meristem number for *OX393-6* grown in 1 N fertilizer was 2 (Fig. 4E), which resembled that of ZH11 with 4 N fertilizer (Fig. 4C). This suggests that OsmiR393 overexpression has the same effect on axillary bud growth as growth with 4 N fertilizer, in ZH11 plants, and that both N fertilizer supply and OsmiR393 expression promote outgrowth of the axillary bud. OsmiR393 expression in rice organs was also analysed by qRT–PCR (Fig. 4G). The results showed that OsmiR393 was expressed in all rice organs, and at a higher level in the roots and booting panicle, indicating that OsmiR393 might play a role in booting panicle development and lateral root growth. To investigate whether OsmiR393 is expressed in the axillary meristem, *in situ* RNA hybridization experiments were performed (Fig. 4F). The results showed that OsmiR393 was strongly expressed in the lateral axillary meristem (Fig. 4F). These results demonstrate that N fertilizer and OsmiR393 overexpression promote outgrowth of axillary buds, and that OsmiR393 is involved in axillary bud formation.

### N fertilizer facilitates rice tillering through preventing the degradation of OsIAA6

TIR1 and AFB2 are two members of the SCF complex, which degrades auxin signalling repressors, to ensure correct auxin signaling^45^,^48^. To determine which proteins are degraded by the SCF^TIR1^ complex, a construct with cMYC fused to OsTIR1 was generated and transformed into ZH11. Subsequently, a co-immunoprecipitation assay was employed and the precipitated proteins were analysed by mass spectrometry. We found that levels of OsIAA6, a protein belonging to the AUX/IAA class of auxin signalling repressors, changed tremendously between *35S:cMYC–TIR1* and *OX393-6* plants. *OsIAA6* was reported to enhance rice drought tolerance and regulate tiller outgrowth^49^. Furthermore, AUX/IAA proteins are a substrate of the SCF^TIR1^ complex^50^. We suspected that OsmiR393 might block degradation of OsIAA6 by the OsTIR1 and OsAFB2-mediated SCF^TIR1^ complex to control rice tillering. To investigate whether *OsIAA6* expression changed following altered OsmiR393 expression, or in N-treated ZH11, we generated a construct containing an OsIAA6 full-length protein fused to a MYC epitope tag. This construct was transformed into protoplasts of OsmiR393-overexpressing rice plants, *OsMIR393* mutants, and N-treated ZH11. Western blot analysis showed that OsIAA6 was remarkably increased in the three OsmiR393 overexpressing lines (Fig. 5A), and in ZH11 with elevating N levels (Fig. 5B), but was reduced in *OsMIR393* mutants (Fig. 5C). These results suggest that OsmiR393 might affect auxin signal transduction through regulation of OsIAA6 accumulation.

## Discussion

Availability of N fertilizer strongly affects rice tillering^7^, which is a major determinant of grain yield. In the past decades, several key regulators of rice tillering have been cloned and characterized, including *MOC1*^17^, *TAD1*^51^, *LAX1* and *SPA*^18^, *OsTB1*^52^, OsmiR156^20^, and genes in the strigolactone signalling pathway^53^,^54^,^55^,^56^. However, the mechanisms through which N fertilizer contributes to rice tiller formation remains elusive. In this study, we revealed a novel role for OsmiR393 in affecting rice tillering in response to N fertilizer. Our results show that adding N fertilizer increases the tiller number in rice (Fig. 1B). This is achieved by enhancing the initiation and activation of tiller bud outgrowth (Fig. 4A–D). High N fertilizer causes OsmiR393 accumulation in tiller bud (Figs 1A and 4F; Supplemental Fig. S1). OsmiR393 accumulation then decreases the transduction of auxin signalling (Figs 3 and 5), resulting in decreased sensitivity to auxin in the tiller buds (Supplemental Fig. S3). Consequently, dampening the response to auxin signalling in the tillers with high N fertilizer might promote initiation and outgrowth of the tiller bud.

### OsmiR393 responses to N fertilizer

*Arabidopsis* miR393 is induced by nitrate, and the nitrate-responsive miR393/AFB3 regulatory module mediates root system architecture^8^. In this study, the addition of N fertilizer also caused the accumulation of OsmiR393 in rice seedlings (Fig. 1A; Supplemental Fig. S1). This result suggests that in plants, a similar mechanism has been adopted in monocots and dicots, to respond to environmental changes in N levels. Over time, expression of its target genes (*OsTIR1* and *OsAFB2*) was repressed (Fig. 3B). This suggests that OsmiR393 is also involved in the response to exogenous N in rice. However, unlike *Arabidopsis* miR393, wherein expression induced by NO_3_^−^ was specifically localized to the root^8^, the expression of OsmiR393 in rice was induced by NH_4_NO_3_ in the leaf and root tissue (Figs 1A and 4F). This discrepancy between rice and *Arabidopsis* might be due to different N treatments, or it might imply that there are functional differences for miR393 between dicots and monocots.

### OsmiR393 affects rice tillering

miR393 is a plant-conserved miRNA that participates in many processes during plant development. Recent studies have demonstrated that miR393 plays a role in the establishment of root system architecture in response to nitrate^8^, the regulation of leaf development^28^, and auxin signaling^57^, and is involved in antibacterial resistance in response to pathogens^27^. In this study, overexpression of OsmiR393 resulted in an increased number of tillers, compared to that in wild type plants (Fig. 1), which was similar to previous findings^39^. In contrast, an *OsMIR393* mutant produced fewer tillers than wild type rice (Fig. 2). Furthermore, supplementation with N did not rescue this defect in *OsMIR393* mutants. These results suggest that *OsMIR393* is a regulator that participates in modulating rice tillering.

In addition, OsmiR393 altered tillering in response to N Fertilizer. OsmiR393 was induced by NH_4_NO_3_ treatment in rice (Fig. 1A), and was highly expressed in the bud meristem (Fig. 4F). When OsmiR393 was overexpressed, the increase in tiller numbers induced by N fertilizer (Supplemental Fig. 2) was not as high as that observed in wild type rice (Fig. 1B,C). However, with the same N level, OsmiR393-overexpressing rice produced more tillers than wild type rice (Fig. 1E,B compared to Supplemental Fig. S2). In contrast, the mutation of *OsMIR393* resulted in fewer tillers than that in wild type with high and normal N fertilizer levels (Fig. 2). These results suggest that *OsMIR393* is involved in the regulation of rice tillering in response to N fertilizer. Rice varieties exhibit high diversity with respect to N response. According to the degree of responsiveness, rice varieties were classified into two groups, less-responsive varieties (such as, ZH11, QZL2, and Balila) and highly-responsive varieties (such as Minghui 63 and Nanjing 6)^58^. Highly-responsive rice varieties tend to produce more tillers than less-responsive varieties under the same level of N fertilizer^58^. OsmiR393 expression was compared (Supplemental Fig. S4) in these two groups. However, the expression of OsmiR393 was not obviously different. Different *DEP1* alleles confer variable N responses among less responsive and highly responsive rice cultivars, and *DEP1* has been the subject of artificial selection during *Oryza sativa* spp. *japonica* rice domestication^58^. This result might imply that OsmiR393 is a general regulator of rice tillering that responds to N fertilizer, but is not involved in the N-response variability observed among different rice varieties.

### OsmiR393 affects auxin signal transduction in response to N fertilizer treatment

Plants synthesize auxin in the growing apex, wherein most of their energy is expended^59^. Auxin flows from the stem towards the root, basipetally, a phenomenon known as polar auxin transport (PAT). This PAT inhibits axillary bud growth in terms of apical dominance^60^. It is well known that plants establish their architecture and apical dominance through the effects of PAT.

In this study, we revealed that N-fertilizer promotes OsmiR393 accumulation (Fig. 1A) and decrease expression of *OsTIR1* and *OsAFB2* (Fig. 3B), and increases tiller number (Fig. 1B) in wild type ZH11 rice. This N-promoted tillering is mimicked by OsmiR393 overexpression (Fig. 1E). Moreover, high N-treatment in wild type ZH11 and overexpression of OsmiR393 resulted in a decrease in OsmiR393-target gene (*OsTIR1* and *OsAFB2*) expression (Fig. 3). The reduced expression of *OsTIR1* and *OsAFB2* might attenuate auxin perception and signalling, and alleviate the effects of apical dominance. TIR1 and AFB2 are two important substrate recognition subunits of the well-known SKP1-CULLIN-F-box (SCF) ubiquitin ligase complex, which functions in auxin perception to promote the 26S proteasome-mediated degradation of Aux/IAA transcriptional repressors^47^. In addition, the response of *MIR393* to environmental signals is conserved across plant species, through the auxin signalling pathway^61^,^62^. It has been shown that auxin cannot enter axillary meristem cells^11^; however, auxin from the top buds could inhibit export of auxin that is synthesized in the axillary buds^11^,^12^. This would increase the auxin concentration in the axillary buds, and inhibit bud outgrowth. High OsmiR393 accumulation after N treatment (Fig. 1A) might decrease sensitivity in the axillary buds to high levels of auxin.

We proved that OsmiR393 overexpression and N treatment prevents degradation of *OsIAA6* (Fig. 5), which is an important regulator of axillary bud formation^49^,^63^. This strongly supports the hypothesis that OsmiR393 influences axillary bud formation following N fertilizer treatment through auxin signal transduction. Auxin signalling components have been conserved throughout land plant evolution, and have evolved to control specific developmental processes^32^. Plant genomes encode large numbers of F-box proteins (FBPs), and there are approximately 700 FBPs in *Arabidopsis*^64^. The role of miR393/TIR1/AFBs in the plant’s response to auxin represents a ubiquitous model for adaption and acclimation. Therefore, we concluded that N fertilizer promotes OsmiR393 accumulation, which interferes with auxin signalling, finally triggering tiller production in rice.

N fertilizer can promote cytokinin biosynthesis/signalling and stimulates rice to produce more tillers^65^,^66^. There is evidence to suggest that exogenous application of N fertilizer can promote cytokinin biosynthesis^67^ and stimulate lateral bud outgrowth^68^. In *Arabidopsis*, the expression of *AtIPT3* and *AtIPT5* is in response to N^69^,^70^; these genes are key determinants of CK biosynthesis that respond to rapid changes in NO_3_^−^ availability. Our present results showed that OsmiR393 also responds to N fertilizer, interferes with auxin signalling, and attenuates apical dominance, ultimately leading to tiller production in rice. We did not analyse whether these two N-mediated tillering pathways overlap. Thus, the effect of N fertilizer on rice tillering might be through these two pathways, or in parallel, or through other undiscovered pathways. This also explains why exogenous N-treated *OsMIR393* mutants still produced more tillers than wild type plants in normal N conditions (Fig. 2B,C,E,F).

## Materials and Methods

### Plant materials and growth conditions

*Oryza sativa* japonica Zhonghua11 (ZH11) was used as the wild type and the source for transgenic plants. Plant materials used in this study were ZH11, three OsmiR393 overexpressing lines (*OX393-6*, *OX393-10*, and *OX393-31*)^39^, three *OsMIR393* knock-out lines (*CRP-9*, *CRP-15*, and *CRP-18*), and the distinct N responsive varieties (QZL2, Balila, Minghui 63, and Nanjing 6)^58^. Seeds were dried at 37 °C for 24 h before germination at 25 °C, and were supplemented with water in the dark for 48 h. For phenotypic observations, germinated seeds were grown under controlled field conditions or in boxes filled with sand supplemented with hydroponic medium. For nutrient treatments, plants were grown in hydroponic cultures using the International Rice Research Institute (IRRI) liquid culture medium recipe (IRRI nutrient solution)^38^. Except for modified NH_4_NO_3_, all nutrients were kept at the same concentration in all cultures. The NH_4_NO_3_ content was based on that of the IRRI nutrient solution, which was designated as the normal level; other N fertilizer levels were based on this concentration. For example, NH_4_NO_3_ content in 4-fold N fertilizer was quadruple that of the normal level. For paddy culture, seeds were germinated and sown on soil and then conventionally managed.

### Vector construction for rice transformation

To generate the *cMYC-OsTIR1* construct, the coding region of *OsTIR1* was amplified by PCR, cloned into the pEASY–Blunt vector (Transgene, China), and subcloned into the pE3n vector^71^. The *cMYC-OsTIR1* fragment was then inserted into the pCAMBIA2300 vector downstream of the maize (*Zea mays*) *Ubiquitin* promoter after *Kpn*I and *Bam*HI digestion. To generate the *cMYC-OsIAA6* construct, the coding region of *OsIAA6* was amplified by PCR, cloned into the pEASY-Blunt vector (Transgene, China), and subcloned into the pE3n vector. The *cMYC-OsIAA6* fragment was then inserted into the pCAMBIA2300 vector downstream of the maize (*Zea mays*) *Ubiquitin* promoter after *BamH*I and *Not*I digestion. All of the primers used to generate the aforementioned constructs are listed in Supplemental Table S1, and all of the constructs were confirmed by sequencing. The constructed vectors were transformed by *Agrobacterium tumefaciens* strain EHA105. Wild type ZH11 calli were used as the recipients for *Agrobacterium*-mediated transformation as described^72^.

### RNA extraction and quantitative RT–PCR

Total RNA was extracted using an RNA extraction kit (Invitrogen, China) and digested with DNase I (Takara, China) according to the manufacturers’ instructions. The RNA quality and integrity were analysed by agarose gel electrophoresis and the RNA concentration was determined using a biophotometer (METASH, B–500, China). cDNA was synthesized from total RNA using AMV Reverse Transcriptase (Promega, China). Small RNA was extracted using an RNAiso kit for small RNA (Takara, China) and digested with DNase I (Takara, China) according to the product manuals. Reverse transcription was performed with a cDNA Synthesis Kit (Promega, China) in combination with a stem-loop RT-PCR technique^73^. Quantitative RT-PCR was performed on a 7500 RT-qPCR system (Applied Biosystems, USA) with SYBR Green Real-time PCR Master Mix (Toyobo, China) according to the manufacturer’s instructions. Gene expression was normalized to that of rice *ACTIN1*. Primers used for qRT-PCR are presented in Supplemental Table S1.

### Small RNA gel blot assay

Small RNA gel blot analysis was performed as described in Liu *et al*.^74^. Briefly, total RNA was extracted from rice seedlings, and total RNA samples (approximately 20 μg) were separated on denaturing 15% polyacrylamide gels and transferred electrophoretically to Hybond-N^+^ membranes (http://www.gelifesciences.com/). The gel was stained with ethidium bromide before transfer to confirm equal loading. Hybridizations were performed at 42 °C in PerfectHyb Plus buffer with DNA oligonucleotide probes labelled by T4 polynucleotide kinase (New England Biolabs, https://www.neb.com/). Hybridization signals were detected with a phosphorimager (GE Healthcare Life Sciences, http://www.gelifesciences.com). The sequences of the probes are provided in Supplemental Table S1.

### Tissue sectioning and *in situ* hybridization

Seedlings of ZH11 plants were grown with different N fertilizer levels, and *OX393-6* was grown with normal levels of N fertilizer for 15 d, after which SAM tissues and axillary buds were fixed and sectioned at a thickness of 7 mm. The sections were then stained with toluidine blue for light microscopic analysis (Zeiss, Germany). *In situ* hybridization was performed as described^75^. The OsmiR393 probe was synthesized and labelled with digoxigenin. Shoots containing SAM and axillary meristems were used for hybridization assays. Slides were photographed under a microscope (Zeiss, Germany).

### CRISPR-mediated mutation of *OsMIR393*

An sgRNA: AAGGATCAATGCGATCCCTTTGG was designed to target OsmiR393. The sgRNA was inserted into the *Aar*I site of the p2300-rCas9-U3-gRNA vector, which contains a rice-codon optimized Cas9 driven by a 2× 35S promoter^42^, and the sgRNA was activated by an *OsU3* promoter. The primer used for cloning is shown in Supplemental Table S1 (CRISPR-F and CRISPR-R). After introducing the CRISPR/Cas plasmid into rice varietal ZH11 through *Agrobacterium*-mediated transformation^72^, the T_0_ generation mutants were screened with G418 (Invitrogen). All regenerated T_0_ transgenic plants were genotyped using the primer (CRISPR-g-F and CRISPR-g-R) to select positive transgenic lines, and a small RNA gel blot analysis was performed to subsequently detect OsmiR393 expression and confirm the knock-out of OsmiR393 in mutated lines.

### Co–IP and mass spectrum analysis

To validate the protein interaction profile of OsTIR1 *in vivo*, a Co-IP assay was employed using an immunoprecipitation kit (Sigma-Aldrich, China). The cDNA for *OsTIR1* was amplified using the primers cMYC-TIR1-F and cMYC-TIR1-R. Amplified cDNA was inserted into pRT107–6XMyc between the *BamH*I and *Kpn*I sites to generate the expression vector 2×35S:6XMyc-OsTIR1. The co–immunoprecipitation procedure was performed in accordance with the manufacturer’s instructions using an anti-c-Myc antibody (Sigma-Aldrich, China). Mass spectrum analysis was performed by the Boxin biotechnology company in Guangzhou. Primers used are listed in Supplemental Table S1.

### Protoplast isolation and western blot analysis

To generate the cMYC-OsIAA6 sequence, the *OsIAA6* coding sequence was amplified with the gene specific primers, cMYC-OsIAA6-F and cMYC-OsIAA6-R containing the restriction enzyme sites *BamH*I and *Not*I, and ligated into a rice transformation vector for constitutive expression. Constructs were introduced into rice protoplasts of 2-week seedlings as previously reported^76^. The generated constructs were then transfected into the isolated protoplasts using polyethyleneglycol-mediated transformation^76^. To examine OsIAA6 protein expression, a western blot was performed as described in Niu *et al*.^77^.

### Tiller bud and callus induction with NAA treatments

Five-leaf seedlings were sprayed with 5 mg/L of NAA, and the length of tiller buds was measured as indicated. Callus induction with 2 mg/L of NAA was performed as previously described^39^.

### Tiller count and data analysis

Rice plants were planted in previously described conditions and grown for approximately 30 days before the headings and tillers were counted; all data were analysed in Excel using a *t*–test.

Sequence data from this study can be found in the GenBank/EMBL data libraries under accession numbers *OsAFB2* (LOC_Os04g32460), *OsTIR1* (LOC_Os05g05800), *OsIAA6* (LOC_Os01g53880), and *ACTIN1* (LOC_Os03g50885).

## Additional Information

**How to cite this article**: Li, X. *et al*. MicroRNA393 is involved in nitrogen-promoted rice tillering through regulation of auxin signal transduction in axillary buds. *Sci. Rep.* **6**, 32158; doi: 10.1038/srep32158 (2016).

## Supplementary Material

Supplementary Information
