# Supplementary material for: MicroRNA393 is involved in nitrogen-promoted rice tillering through regulation of auxin signal transduction in axillary buds
Source: Sci Rep. 2016 Aug 30;6:32158. doi: 10.1038/srep32158 (PMC5004122; doi:10.1038/srep32158)
Supplement: Supplementary Information [file srep32158-s1.pdf]

Supplementary information

MicroRNA393 is involved in nitrogen-promoted rice tillering through regulation of auxin signal transduction in axillary buds

Xiang Li[1], Kuaifei Xia[1], Zhen Liang[2], Kunling Chen[2], Caixia Gao[2] & Mingyong Zhang[1*]

[1]Key Laboratory of South China Agricultural Plant Molecular Analysis and Genetic Improvement & Guangdong Provincial Key Laboratory of Applied Botany, South China Botanical Garden, Chinese Academy of Sciences, Guangzhou 510650, China. [2]State Key Laboratory of Plant Cell and Chromosome Engineering, Institute of Genetics and Developmental Biology, Chinese Academy of Sciences, Beijing 100101, China. Correspondence and requests for materials should be addressed to M.Y. (email: zhangmy@scbg.ac.cn)

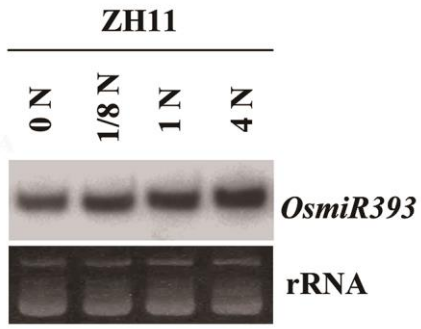

Figure S1. Supply of NH_4NO_3 triggered OsmiR393 accumulation. Small RNA gel blot analysis of OsmiR393 expression level in ZH11 under elevated NH_4NO_3 levels.

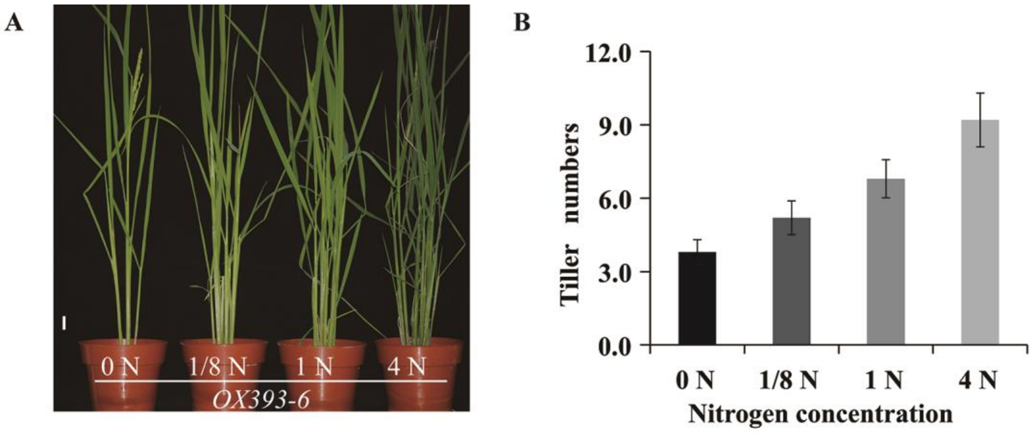

Figure S2. OsmiR393 overexpressing line (*Ox393–6*) treated with NH_4NO_3, exhibit increased tiller number, which is not to the extent of ZH11 in Fig. 1. B-C, Tillers of *OX393–6* treated with gradient NH_4NO_3 concentrations. Bar = 3 cm. **B**, Statistical analysis of (A) by *t*-test, Vertical bars indicate standard error deviation. Three individual repeats were performed.

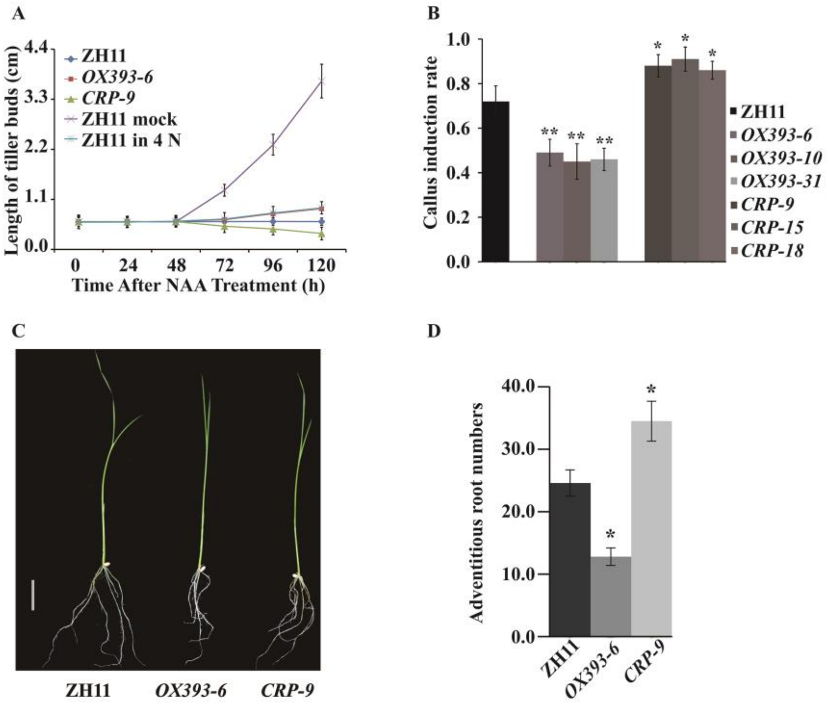

Figure S3. OsmiR393 involved in auxin signaling transduction.

A, Effect of NAA treatment on the outgrowth of rice tiller buds at the third leaf axils. Error bars represent mean ± standard error (n = 20). The NAA spray concentration is 5 mg L^{-1}. **B**, Callus induction rate from seeds of ZH11, OsmiR393–overexpressing lines (*OX393*), and *OsMIR393* knock–out mutants (*CRP*) with 2 mg L^{-1} NAA. **C**, Root architecture of ZH11, *OX393–6*, and *CRP–9* under 1 N level. Bar = 3 cm. **D**, Statistical analysis of adventitious root numbers in ZH11, *OX393–6*, and *CRP–9*. Eighteen 7-day-old seedlings of each line were used for adventitious roots number counting. The error bar represents mean ± SE. The asterisk indicates significant differences (* P ≤ 0.05 and ** P ≤ 0.01) compared with ZH11 by *t*-test.

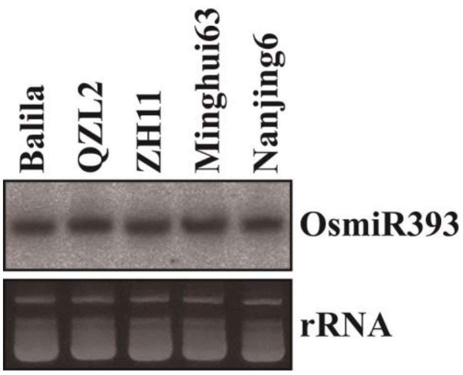

Figure S4. OsmiR393 expression level in N less–responsive and highly sensitive rice varieties. A small RNA gel blot assay was performed to detect OsmiR393 expression in N less–responsive varieties (ZH11, QZL2, and Balila), and in highly sensitive varieties (Minghui 63 and Nanjing 6). The total rRNA was used as a loading control.

Supplementary Table S1 Primer sequences used in this study

Name	Sequence (5' - 3')
393-q-F	GACTGTCCAAAGGGATCGCATT
393-q-R	GTGCAGGGTCCGAGGTATTC
Actin-q-F	CGGTGTCATGGTCGGAAT
Actin-q-R	GCTCGTTGTAGAAGGTGT
U6 q-F	CGATAAAATTGGAACGATACAGA
U6 q-R	ATTTGGACCATTTCTCGATTTGT
U6-RT	ATTTGGACCATTTCTCGATTTGT
OsAFB2- q-F	GAGCGGGATGGTAGCAATGAAATG
OsAFB2- q-R	CCGAGATAAGGGAGGCACACCAAC
OsTIR1-q-F	TCCAGGTGCTCCGCCTCGTCTCCT
OsTIR1-q-R	CCGGGAAGAGGCTGAGCCAATGAA
393-RT	GTCGTATCCAGTGCAGGGTCCGAGGTATTCGCACTGGATACGACGAT
36080-q-F	TCAAGGAGGACCAGCAGTTCACCA
36080-q-R	GAGGACACCCGTGCTGACCAAAGT
41010-q-F	GCGGAGGTGGAGGCGGGAAGG
41010-q-R	CGCGAGGGAGACGAGGTGGTTG
39790-q-F	TATGGTATGCCCGGTGGAAGAAGT
39790-q-R	ATACTGCTGCCTCAGGATGATTGG
52320-q-F	TGCTTCCCCTACCACCGTCACCAC
52320-q-R	GCGCACTCTTCCACCTTCCCATTG
58734-q-F	TTGCTTCCGCCTCCTCATCTTCCT
58734-q-R	GCCGTGCGAACCATATTCCTTTGA
393 probe	GATCAATGCGATCCCTTTGGA
CRISPR-F	GGCAAAGGATCAATGCGATCCCTT
CRISPR-R	AAACAAGGGATCGCATTGATCCTT
CRISPR-g-F	CCTCACCAAAATACGAAAGAG
CRISPR-g-R	CAGTCACGACGTTGTAAAAC
cMYC-TIR1-F	GGATCC ATGGGGCGCGGCGGCTCGCG
MYC-TIR1-R	GGTACC CTAGAGTCCATGCATTTCCA
cMYC-OsIAA6-F	GGATCC ATGGAAGAAGGGTCCAACAA
cMYC-OsIAA6-R	GCGGCCGC GACCCTAGCAGTAGCTCCAA

Supplementary Table S2 Statistical analysis data of axillary meristem between ZH11and *OX393-6*

	0 N (NAM/TP)	1 N (NAM/TP)	4 N (NAM/TP)	8 N (NAM/TP)
ZH11	2/20	9/20	17/20	20/20
OX393-6	/	13/20	/	/

ZH11 and *OX393-6* were sown and cultivated in hydroponic solution according to IRRI nutrient solution[38] under various levels of NH_4NO_3 fertilizer treatment whereas *OX393-6* was under normal (1 N) NH_4NO_3 fertilizer concentration. After 14 days, 20 samples were sectioned to observe axillary meristems both in ZH11 and in *OX393-6*. NAM: observed numbers of axillary meristem, TP: total plants sampled.
